# Lacto-fermented vegetables as dynamic food ecosystems: emerging mechanisms along the microbiome–gut–brain axis

**DOI:** 10.3389/fnut.2026.1903488

**Published:** 2026-08-12

**Authors:** Gonza B. Ngoumou, Steven Ngandeu Schepanski, Fatemeh Nejati, Alina Diedering, Noemie Cassagnau, Carla Beyrow, Wisnu Adi Wicaksono, Gabriele Berg, Georg Seifert

**Affiliations:** 1 Charité Competence Center for Traditional and Integrative Medicine (CCCTIM), Charite - Universitatsmedizin Berlin, Berlin, Germany; 2 Department of Biotechnology, Technische Universität Berlin, Berlin, Germany; 3 Institute of Environmental Biotechnology, Graz University of Technology, Graz, Austria; 4 Department of Microbiome Biotechnology, Leibniz Institute for Agricultural Engineering and Bioeconomy, Potsdam, Germany; 5 Department of Pediatrics, University of São Paulo, São Paulo, Brazil

**Keywords:** bioactive metabolites, fermentation, fermented foods, gut microbiome, lactic acid bacteria, lacto-fermented vegetables, microbiome-gut-brain axis

## Abstract

Lacto-fermented vegetables (LFVs) are traditional foods produced through the microbial transformation of plant materials by lactic acid bacteria, with additional contributions from yeasts and other microorganisms. Fermented foods have been increasingly investigated for their potential modulation of the microbiome-gut-brain axis. This research has mainly focused on dairy fermentations, leaving LFVs largely underexplored. This mini review examines LFVs and their emerging mechanisms within the microbiome-gut-brain axis (MGBA). It emphasizes their character as evolving microbial ecosystems and as sources of bioactive metabolites. During the fermentation process, ecological succession drives the production of a variety of compounds, including organic acids, amino acids and lipid derivatives, transformed phytochemicals, structural microbial components, and micronutrients. Many of these compounds have plausible connections to MGBA routes. These links include modulation of the intestinal barrier, local and systemic immune responses, enteroendocrine signaling, and autonomic/vagal pathways. Particular focus is given to the small intestine as an early signaling interface between metabolites and host physiology. Scientific evidence of the beneficial effects of LFVs on human health is limited. Existing studies show improvement in gastrointestinal symptoms, cardiometabolic risk factors and gut microbial function. Studies connecting LFVs to neurological and psychological health are lacking. We propose understanding LFVs as complex food ecosystems whose effects on host physiology arise from interactions among microorganisms, metabolites, environmental conditions, and host responses. Future research integrating food multi-omics, microbiome analyses, immune profiling, neuroendocrine measurements, and clinical outcomes may help clarify the role of LFVs in MGBA and support the development of targeted fermentation-based nutritional strategies for intestinal, immune, and neuropsychological health.

## 1 Introduction

Fermented foods are characterized by the desired growth of microorganisms transforming the food matrix over time (1). Food fermentation has been used by humans for millennia (1, 2). Campbell-Platt et al. estimated the proportion of fermented foods worldwide in 1994 at around one-third of overall food consumption (2). The benefits of preservation, increased food safety and digestive efficiency, and, in some cases, alcohol content likely contributed to the early use of fermentation technologies (2, 3). A large variety of foods are fermented all around the world, including dairy, fruits, vegetables, leaves, cereals, tubers, meat, fish and mushrooms, involving an even larger variety of specialized microorganisms (4).

Despite this diversity, common principles of community assembly can be identified across fermented foods (5, 6). Community composition is determined by dispersal (how microorganisms reach an environment), selection (environmental pressures), diversification and ecological drift, with dispersal and selection being especially useful in the context of food fermentation (5, 6). In fermented foods, dispersal includes the resident microorganisms present in the raw food, the surrounding environment, as well as starter cultures. Previous work has, for instance, shown that different crop management practices can shape the plant microbiome (7). Temperature, salinity, acidity, oxygen availability, nutrient composition, and the activity of bacteriophages, yeasts, and fungi are important environmental selection factors in fermented foods (4, 5, 8). Interestingly, selective pressures and substrate seem to outweigh geographic influences, as similar microbial communities are regularly observed in comparable fermented products across several continents (4, 5).

Existing reviews have detailed how fermented foods serve as carriers for beneficial microbes and metabolites and can modulate the central nervous system via the microbiome-gut-brain axis (MGBA) (9–13). Several reviews also focused on the potential of fermented foods to moderate neurologic and psychiatric health conditions such as depression and anxiety or neurodegenerative diseases (10, 11, 14).

Until now, most research linking fermented foods to the MGBA has focused on dairy ferments such as yogurt and kefir. Lacto-fermented vegetables (LFVs) offer a distinct profile characterized by plant- and soil-derived starter cultures and the development of plant-derived bioactive metabolites, including transformed phytochemicals and dietary fiber (12, 13). Throughout diverse vegetable fermentations, the microbial communities are typically dominated by lactic acid bacteria (LAB), particularly species of the genera *Lactobacillus, Leuconostoc, Weissella, Pediococcus*, and *Lactococcus* (12, 15, 16).

**Figure 1:**
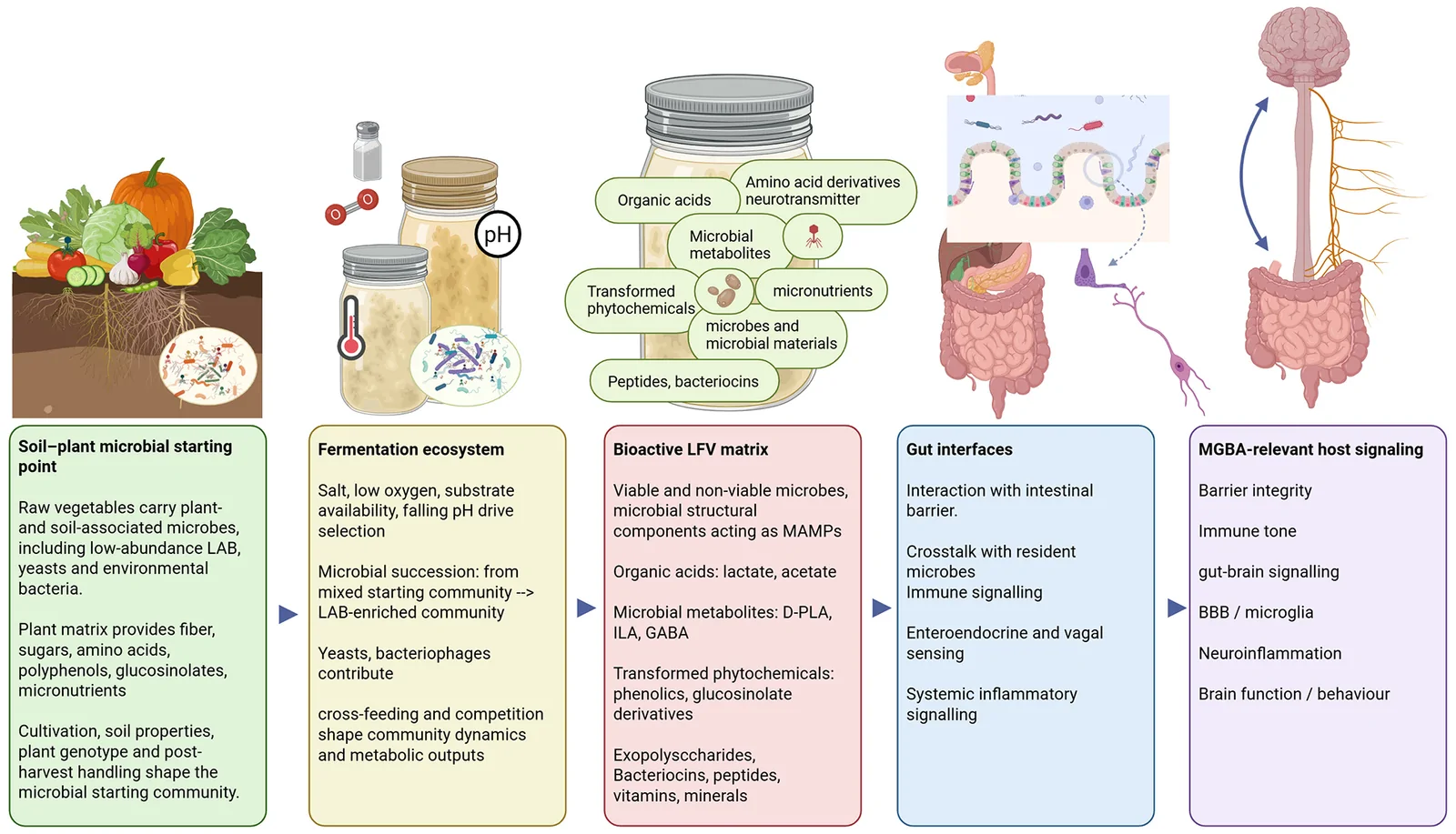
Lacto-fermented vegetables as dynamic food ecosystems along the microbiome–gut–brain axis. Raw vegetables carry plant- and soil-associated microorganisms and plant substrates that enter fermentation. Salt, low oxygen, substrate availability and falling pH select for lactic acid bacteria (LAB), while yeasts, bacteriophages, cross-feeding and microbial competition shape microbial succession and metabolic output. This process generates a bioactive LFV matrix containing viable and non-viable microbes, microbial structural components, organic acids, microbial metabolites such as D-PLA, ILA and GABA, transformed phytochemicals, exopolysaccharides, bacteriocins, peptides, vitamins and minerals. After ingestion, these components may interact with the intestinal barrier, resident microbiota, mucosal immune system, enteroendocrine cells and vagal pathways. Through overlapping barrier, immune, endocrine, neurovascular and neural routes, LFV-derived signals may plausibly contribute to MGBA-relevant host signaling. Direct LFV-specific evidence for neurological or psychological outcomes in humans remains limited. BBB, blood–brain barrier; D-PLA, D-phenyllactate; GABA, gamma-aminobutyric acid; ILA, indole-3-lactic acid; LAB, lactic acid bacteria; LFV, lacto-fermented vegetable; MAMPs, microbe-associated molecular patterns; MGBA, microbiome–gut–brain axis. Created in BioRender. Ngoumou, G. (2026) https://BioRender.com/v1iyl7v.

This mini review will focus specifically on LFVs and their possible mechanisms of action on the central nervous system (CNS). The particular perspective centers around an understanding of LFVs as complex food matrices that may act dynamically over time through combined direct and indirect pathways (Figure 1).

## 2 Biochemical evolution in LFVs

### 2.1 Microbial succession

The transformation of a raw vegetable into bioactive fermented foods is controlled by a time-dependent microbial and biochemical succession (16, 17). Lacto-fermentation occurs in a low-oxygen environment where salt, acidification, substrate composition and microbial interactions select for specialized LAB (13). Initial communities derive largely from plant- and soil-associated microorganisms. Although LAB may occur at low abundance before fermentation, their presence and succession can be influenced by geographical region, soil properties, farming practices, plant genotype and post-harvest handling (18). Spontaneous LFV fermentation may therefore retain microbial signatures from the agricultural ecosystem, including non-LAB members, before conditions select for acid-tolerant LAB (19). This links LFVs to a broader soil-plant-food-microbial continuum (5, 18, 19).

Early fermentation is frequently dominated by heterofermentative species such as *Leuconostoc mesenteroides* and *Weissella spp*., producing lactate, acetate, CO2 and ethanol. As pH decreases, communities typically move toward more acid-tolerant homofermentative LAB, including *Lactiplantibacillus plantarum* and related *Lactobacilli* (15, 16). This ecological succession shapes the developing metabolic landscape of LFVs (15–17).

### 2.2 Organic acids

Lactate is one of the most abundant metabolites in many LFVs (13, 20, 21). It drives acidification, supporting pathogen suppression (22, 23). Beyond preservation, orally administered lactate has been linked to enteroendocrine effects in humans, including increased GLP-1, insulin, and glucagon, reduced ghrelin, and earlier satiety (13, 21). Lactate can also activate the human HCA1 receptor, which is involved in intestinal barrier integrity and inflammatory signaling (10). In preclinical and in vitro models, lactate exhibited barrier-protective and anti-inflammatory effects and supported the expansion of beneficial taxa such as *Akkermansia muciniphila*, with downstream effects on regulatory T cells (13, 24, 25).

Acetate is produced by LAB and may also arise through cross-feeding (26, 27). It is rapidly bioavailable after ingestion and has been associated with improved fasting glucose in humans (28, 29). Acetate activates the short-chain-fatty acid (SCFA) receptors GPR43 and GPR41, involved in immune regulation, gut hormone synthesis, barrier integrity, and neuronal function (30). Other SCFA, including propionate and butyrate, may also be present in some LFVs or arise after fermentation-derived compounds are further metabolized by resident microbes (31–34).

### 2.3 Amino-acid and lipid-derivatives

LAB can convert amino acids into active metabolites (35). Phenylalanine can be transformed into D-Phenyllactate (D-PLA), which increases in plasma and urine after sauerkraut consumption and acts as a potent agonist of the human HCA3 receptor (36–38). Tryptophan-derived indole-3-lactic acid (ILA), detected in different LFVs, can activate HCA3 and the Aryl hydrocarbon receptor (AhR), a transcription factor involved in barrier regulation and immune signaling (13, 39–41). Interestingly, the human-specific HCA3 receptor may represent an evolutionary adaptation to the habitual consumption of fermented foods, converting microbial signals into immune responses via monocytes and the intestinal epithelium (10, 38). LAB-driven decarboxylation of glutamate can generate gamma-aminobutyric acid (GABA), an inhibitory neurotransmitter (42). Although orally ingested GABA likely has limited direct access to the brain, peripheral GABAergic signaling may influence gut motility, secretion, inflammation and immune tolerance (25, 43, 44). LFVs can also contain other amino acid derivatives, such as 2-hydroxyisocaproic acid and 12,13-DiHOME, with potential relevance to microbial signaling, barrier and immune interactions (45–47).

### 2.4 Transformed phytochemicals

LFVs contain plant secondary metabolites, typically derived from the raw plant (13, 48). LAB enzymes, including ß-glucosidases, can convert glycosides into smaller aglycones with potentially higher bioavailability (49–51). In cruciferous vegetables, fermentation promotes glucosinolate degradation and the formation of indole-derivatives such as indole-3-carbinol and ascorbigen, which may contribute to antioxidant and anti-inflammatory properties (52, 53). Fermentation may also increase total phenolic content by releasing bound phenolic derivative compounds (13, 54). Some phenolic compounds and their metabolites have been associated with metabolic, cardiovascular and neurologic benefits, although LFV-specific human data remain limited (13, 55–58).

### 2.5 Bacteriocins, exopolysaccharides and microbial structural components

LAB produce bacteriocins, antimicrobial peptides that can shape microbial ecology by suppressing competing bacteria (13, 59–61). Exopolysaccharides are another class of fermentation-associated bioactives and may support barrier integrity, community structure and mucosal immune modulation (13, 17, 62, 63).

LFVs also contain non-viable microbial components, including peptidoglycans, cell wall fragments, lipoteichoic acids, CpG DNA and surface proteins (64–66). These microbe-associated molecular patterns (MAMPs) interact with host pattern recognition receptors (PRRs). Those interactions are context-dependent: in balanced settings, microbial sensing can support immune training, whereas in dysregulated states it may drive inflammatory responses (10, 67).

### 2.6 Micronutrients and reduction of anti-nutrient

Fermentation can modify the micronutrient profile of vegetables and fruits. LFV may show increased concentration of B vitamins, including folate, riboflavin, vitamin B12 and vitamin K (13, 68, 69). Effects on vitamin A and vitamin C vary by substrate and fermentation (13, 68, 70). Although some mineral concentrations may decline (e.g., iron, magnesium, calcium), mineral bioavailability can improve through microbial phytase activity, which reduces phytates that otherwise bind cations (13, 71).

### 2.7 Bacteriophages and yeast in LFVs

LFVs contain bacteriophages that co-evolve with bacterial communities and shape succession by infecting LAB and other fermentation bacteria (8, 72, 73). Phage-mediated lysis limits overgrowth and releases microbial material that supports cross-feeding (74, 75). Yeast, including *Saccharomyces spp*. and *Candida spp.*, also participate in carbohydrate metabolism, flavor formation, oxygen availability, nutrient synthesis and microbial succession, thus affecting the functional properties of LFVs (17, 76, 77).

## 3 Direct and indirect modulation of the MGBA: overlapping routes

The microbiome communicates with the brain through bidirectional routes collectively termed the microbiota-gut-brain axis (MGBA) (78, 79). Known mechanisms include changes in gut microbiome ecology, immune and inflammatory signals, intestinal and blood-brain barrier integrity, the hypothalamus-pituitary-adrenal (HPA) axis, neurotransmitters, gut hormones and vagal signaling (78–80).

In the context of LFVs, direct and indirect MGBA effects should be viewed as overlapping rather than separate pathways (Table 1). LFVs deliver microbial components and fermentation-derived metabolites that interact with intestinal barrier, immune, endocrine and neuronal interfaces. Additionally, they provide live-plant-associated microorganisms that can transiently engage with resident communities via cross-feeding and competition (10, 16, 26, 79, 81). Plant-associated microorganisms specific to LFVs may therefore contribute indirectly to MGBA-relevant pathways by modifying the microbial and metabolic starting conditions of fermentation (18, 19).

**Table 1:** LFV-derived bioactive components and plausible MGBA-relevant routes.

LFV-derived component/class	Origin in LFVs	Representative examples	Molecular/cellular targets	Main intestinal interface	Plausible MGBA-relevant route	Current evidence and interpretation
Lactate	Produced as a major end-product of LAB carbohydrate fermentation Contributes to acidification and pathogen suppression	L-lactate, D-lactate depending on microbial species and fermentation conditions	HCA1/GPR81 receptor signaling Epithelial barrier pathways Immune cells Enteroendocrine signaling Resident microbial cross-feeding networks	Small intestine, colon, epithelial barrier, enteroendocrine cells, resident gut microbiota	May support epithelial barrier integrity, reduce inflammatory epithelial injury, influence gut hormone release, and provide substrate for cross-feeding into SCFA-producing pathways. Through barrier, immune and endocrine effects, lactate may indirectly influence MGBA signaling.	Lactate is one of the most abundant metabolites in many LFVs (13, 22, 23). It contributes to acidification and pathogen suppression (23, 24). Oral lactate has been linked to increased GLP-1, insulin and glucagon, reduced ghrelin and earlier satiety in humans (13, 22). Preclinical and *in-vitro* studies support barrier-protective and anti-inflammatory effects (13, 25, 26).
Acetate	Produced by heterofermentative LAB and through microbial cross-feeding	Acetate, acetic acid	FFAR2/GPR43 and FFAR3/GPR41 receptor signaling Immune cells Enteroendocrine cells Barrier pathways Neuronal/metabolic signaling	Intestinal epithelium, mucosal immune system, enteroendocrine cells, systemic circulation	May regulate mucosal immunity, gut hormone synthesis, intestinal barrier function and glucose metabolism. As a rapidly bioavailable SCFA, acetate may contribute to systemic immune-metabolic changes relevant to brain signaling.	Acetate is produced by LAB and can arise through cross-feeding (27, 28). It is rapidly bioavailable after ingestion and has been associated with improved fasting glucose in humans (29, 30). Acetate activates SCFA receptors involved in immune regulation, gut hormone synthesis, barrier integrity and neuronal function (31).
Other SCFAs and SCFA-generating pathways	Some SCFAs may be present in LFVs Others arise after resident gut microbes metabolize LFV-derived lactate, fiber and plant substrates	Propionate, butyrate, lactate-derived cross-feeding products	FFAR2/GPR43, FFAR3/GPR41, GPR109A receptor signaling Histone deacetylases Epithelial cells T cells Microglia-relevant immune pathways	Mainly colon, but some upstream sensing may occur depending on compound availability	Butyrate supports colonocyte energy metabolism and epithelial tight-junction integrity; Propionate can stimulate PYY and GLP-1; SCFAs can shape systemic immune tone and have been linked experimentally to microglial maturation and BBB integrity.	Propionate and butyrate may be present in some LFVs or arise through resident microbial metabolism (32–35). SCFAs regulate barrier function and mucosal immune tone (94, 95) Broader MGBA research links SCFAs to microglial maturation and BBB integrity (82–84).
D-phenyllactate and aromatic amino-acid derivatives	LAB transformation of phenylalanine and other aromatic amino acids during fermentation	D-phenyllactate/D-PLA, 3-phenyllactic acid	HCA3/GPR109B receptor signaling Monocytes Intestinal epithelial cells	Intestinal epithelium, circulating immune cells, mucosal immune interface	May convert microbial fermentation signals into immune responses. HCA3 activation may influence monocyte migration and mucosal immune signaling, thereby contributing to neuroimmune MGBA routes.	LAB can convert phenylalanine into D-PLA (35–37). D-PLA increases in plasma and urine after sauerkraut consumption and acts as a potent agonist of human HCA3 (38). HCA3 may represent an evolutionary adaptation to fermented-food-derived microbial metabolites (10, 38).
Tryptophan-derived indole metabolites	LAB and other microbial transformation of tryptophan-derived compounds; further shaped by plant matrix and gut microbial metabolism	Indole-3-lactic acid/ILA, other indole derivatives	AhR, HCA3 receptor signaling Epithelial barrier pathways Nrf2/NF-κB-related signaling Immune cells	Intestinal epithelial barrier, mucosal immune system, enteroendocrine and neural interfaces	May support barrier integrity, regulate inflammatory tone and shape mucosal immune responses. AhR activation is especially relevant for epithelial defense and immune homeostasis, which may indirectly affect systemic inflammatory inputs to the MGBA.	ILA has been detected in different LFVs (13). It can activate HCA3 and AhR, both relevant to barrier regulation and immune signaling (39–41). ILA-related mechanisms are promising but direct LFV-specific brain-outcome data are lacking.
GABA and other neuroactive amino-acid derivatives	LAB glutamate decarboxylase activity; depends on strain, substrate glutamate, pH and fermentation conditions	Gamma-aminobutyric acid/GABA	GABA receptors in the enteric nervous system and immune cells Gut motility and secretion pathways	Enteric nervous system, epithelial and immune interface, gut motility pathways	LFV-derived GABA is more plausibly a peripheral enteric and neuroimmune signal than a direct CNS neurotransmitter. It may influence motility, secretion, inflammation, immune tolerance and visceral signaling, with possible propagation to the CNS via vagal and spinal afferents.	LAB can generate GABA from glutamate (42). Peripheral GABAergic signaling can regulate motility, secretion, inflammation and oral tolerance pathways (26, 42, 43). Oral GABA likely has limited direct access to the brain, so direct CNS claims should be cautious (97, 98).
HICA, 12,13-DiHOME and other emerging amino-acid/lipid derivatives	LAB amino-acid metabolism and lipid-related microbial transformation during fermentation and gut microbial metabolism	2-hydroxyisocaproic acid/HICA; 12,13-DiHOME	Immune cells Epithelial barrier pathways Microbial community interactions	Gut barrier, mucosal immune system, resident microbiota	May participate in microbial signaling, immune modulation and barrier regulation. Some lipid-derived metabolites may have context-dependent effects on immune tolerance or inflammation.	LFVs can contain HICA and lipid-derived oxylipins such as 12,13-DiHOME (45–47). Their MGBA relevance remains largely indirect and hypothesis-generating.
Glucosinolate-derived compounds from cruciferous LFVs	Fermentation of cabbage, red cabbage, kimchi ingredients and other Brassica vegetables Microbial and plant enzyme-mediated transformation	Indole-3-carbinol, ascorbigen, other glucosinolate degradation products	AhR receptor signaling Antioxidant pathway Inflammatory pathways Xenobiotic-response pathways	Intestinal epithelium, mucosal immune cells, systemic circulation	May influence epithelial defense, antioxidant capacity and immune regulation. This is a plant-specific LFV route that distinguishes vegetable ferments from dairy ferments.	LFVs contain plant secondary metabolites from the raw vegetable matrix (13, 48). In cruciferous vegetables, fermentation promotes glucosinolate degradation and formation of indole derivatives such as indole-3-carbinol and ascorbigen (52, 53).
Phenolic compounds and microbial phenolic metabolites	Release of bound phenolics from plant cell walls LAB enzymatic transformation Further microbial metabolism after ingestion	Flavonoids, phenolic acids, lignans, hydroxytyrosol-like compounds depending on substrate	Antioxidant pathways NF-κB-related inflammatory pathways BBB-relevant transport/metabolism Microglia-related pathways BDNF-related signaling	Intestinal epithelium, systemic circulation, neurovascular interface	May reduce oxidative and inflammatory signaling, modulate immune tone and potentially affect neurovascular or neuroimmune pathways. Some polyphenol metabolites can interact with BBB-related pathways and microglial inflammatory responses in broader research.	LAB enzymes can transform glycosides into smaller aglycones with potentially higher bioavailability (50–52). Fermentation may increase total phenolic content (13, 54). Broader polyphenol literature suggests relevance for BBB permeability, microglia modulation and BDNF-related signaling, but LFV-specific human data remain limited (100–105).
Exopolysaccharides	Secreted by LAB during fermentation; strain- and substrate-dependent	LAB-derived EPS, heteropolysaccharides	Mucus layer Epithelial adhesion Immune receptors Resident microbiota	Mucus layer, epithelial barrier, mucosal immune interface	May influence microbial adhesion, intestinal barrier properties, mucosal immune tone and resident microbial community structure. These effects could indirectly alter inflammatory and metabolic inputs to the MGBA.	Exopolysaccharides are fermentation-associated bioactives with potential effects on barrier integrity, community structure and mucosal immune modulation (13, 17, 62, 63).
Bacteriocins and antimicrobial peptides	LAB secondary metabolism; shaped by strain competition and fermentation ecology	LAB bacteriocins, antimicrobial peptides, many incompletely characterized compounds	Competing bacteria Microbial membranes Gut microbial communities Possible immune interfaces	Fermentation matrix first; later gut microbial ecosystem after ingestion	May shape LFV microbial succession and potentially influence gut microbial ecology after consumption. Effects could be beneficial, neutral or disruptive depending on ecological context and target organisms.	LAB produce bacteriocins that can suppress competing bacteria and shape microbial ecology (13, 59–61). Their MGBA relevance is indirect and mainly mediated through microbial community effects.
Microbe-associated molecular patterns/postbiotic microbial structures	Viable and non-viable bacteria in LFVs Microbial fragments generated during fermentation, storage, digestion or phage-mediated lysis	Peptidoglycans, lipoteichoic acids, CpG DNA, surface proteins, cell wall fragments	PRRs including TLRs and NOD-like receptors Epithelial cells Innate immune cells Antigen-presenting cells	Mucosal immune system, epithelial barrier, Peyer's patches, antigen-presenting cells	Can contribute to immune training, tolerance or inflammation depending on host state, dose, microbial structure and barrier context. Because systemic immune tone is a major MGBA route, this class is highly relevant but context-dependent.	LFVs contain non-viable microbial components and MAMPs (64–66). These interact with PRRs and may support immune training in balanced settings or inflammatory responses in dysregulated states (10, 67).
Viable LAB and transient plant-associated microorganisms	Raw plant microbiome plus fermentation selection LAB succession during fermentation Live microbes entering the gut after consumption	Lactiplantibacillus, Leuconostoc, Weissella, Pediococcus, Lactococcus; other plant-associated microorganisms	Resident gut microbiota Mucosal immune cells Epithelial cells Microbial cross-feeding and competition networks	Lumen, mucus layer, small intestine, colon, immune surveillance sites	Usually transient rather than durable colonizers, but may still alter gut microbial function through cross-feeding, competition, metabolite exchange and immune sensing. This supports the idea that direct and indirect MGBA effects overlap.	LFVs deliver live LAB and associated microbial communities, usually resulting in transient colonization (16, 85). These communities interact with resident microbes through cross-feeding and competition (26, 86). Fermented-food-rich diets show variable effects on gut microbial diversity (87–89).
Yeasts and yeast-associated cross-feeding products	Co-existing microbial populations in some LFVs; contribute to carbohydrate metabolism, oxygen modulation, flavor formation and nutrient synthesis	Saccharomyces spp., Candida spp.; ethanol, aroma compounds, vitamins and other metabolites depending on species	LAB–yeast interaction networks Microbial nutrient exchange Possible epithelial and immune interfaces	Fermentation ecosystem; later gut microbial interface uncertain	Yeasts may shape fermentation trajectory, oxygen availability, LAB succession and metabolite formation, thereby indirectly modifying the host-relevant LFV matrix.	Yeasts contribute to carbohydrate metabolism, flavor formation, oxygen availability, nutrient synthesis and microbial succession in fermented vegetables (17, 76, 77). Their LFV-specific MGBA relevance remains underexplored.
Bacteriophages and phage-derived microbial material	Co-evolution with LAB and other bacterial communities during fermentation; lysis of susceptible strains	LAB phages Phage-mediated bacterial lysates Released microbial nutrients and MAMPs	Bacterial populations Microbial community succession Released microbial fragments Cross-feeding networks	Fermentation matrix; possible downstream gut microbial interface	Phages may regulate bacterial succession, prevent overgrowth, release microbial material for cross-feeding and modify the pool of immunologically active microbial fragments. MGBA relevance is indirect but fits the ecosystem framing.	LFVs contain bacteriophages that co-evolve with bacterial communities and shape succession by infecting LAB and other fermentation bacteria (8, 72, 73). Phage-mediated lysis can limit overgrowth and release microbial material that supports cross-feeding (74, 75).
Micronutrients modified by fermentation	Microbial synthesis, degradation or altered bioavailability during fermentation	Folate, riboflavin, vitamin B12, vitamin K forms, vitamin A and precursors, vitamin C, minerals	Host metabolic pathways Immune function Neuronal metabolism Antioxidant systems	Absorptive epithelium, especially small intestine	May indirectly support immune, metabolic and neurophysiological function, but effects depend strongly on substrate, fermentation conditions, baseline diet and bioavailability.	Fermentation can modify B vitamins, vitamin K, vitamin A precursors, vitamin C and mineral profiles in vegetables and fruits (13, 68–70). This is nutritionally relevant but not a specific MGBA mechanism.
Reduced anti-nutrients and altered mineral bioavailability	Microbial phytase activity and plant matrix degradation during fermentation	Reduced phytates Altered bioavailability of iron, zinc, magnesium, calcium and other minerals	Mineral absorption pathways Host metabolic and immune function	Small intestinal absorption	Improved mineral bioavailability could indirectly support immune and neurophysiological function, but this route is supportive rather than MGBA-specific.	Mineral concentrations may change during fermentation, while microbial phytase activity can reduce phytates that otherwise bind cations and reduce bioavailability (13, 68, 71).
Whole fermented matrix/metabolite consortium	Emergent property of microbial succession, plant matrix degradation, microbial metabolism, microbial lysis and metabolite accumulation	Complete fermented product pasteurized or unpasteurised Microbe-free supernatants	Multiple simultaneous targets: epithelial barrier, immune cells, microbiota, GPCRs, AhR, PRRs, enteroendocrine cells, etc	Barrier and mucosal immune interface, enteroendocrine cells, resident microbiota	The whole matrix may act more strongly than isolated single metabolites, suggesting additive, synergistic or ecological effects. This supports the systems framing of LFVs as complex functional ecosystems rather than single-compound interventions.	Several studies suggest that fermentation-derived metabolites can drive immune and barrier effects even without live microbes (10). Fermented cabbage matrix protected Caco-2 barriers better than single metabolites (47). Fermented vegetable brine and microbe-free supernatants showed comparable barrier and microbiota effects (25). Pasteurized and unpasteurized sauerkraut improved IBS symptoms similarly (91). LFVs may alter the gut metabolome more strongly than the microbiome (92).

At the CNS level, the most plausible LFV-related mechanisms are indirect neuroimmune and neurovascular pathways. Microbial metabolites, such as SCFAs, regulate microglial maturation, homeostasis and function, linking intestinal microbial metabolism and CNS innate immune tone (81, 82). Gut microbiota and SCFA have also been implicated in blood-brain-barrier integrity (81, 83). Although these mechanisms are not LFV-specific, they offer a framework through which LFV-derived organic acids, cross-feed-derived SCFAs, indole derivatives and microbial structural components may influence brain-relevant physiology (84).

LFVs and other fermented foods deliver live LAB and associated microbial communities, usually resulting in transient colonization (16, 85). These transient communities interact with resident microorganisms through cross-feeding or competition (26, 86). Studies have reported variable effects of fermented-food-rich diets on gut microbial diversity (87–89). Additionally, viable and non-viable microbial components can act as MAMPs recognized by the immune system, possibly supporting immune tolerance or inflammation, depending on context (66, 90).

Several studies suggest that metabolites produced during lactic acid fermentation may be sufficient to drive gut immune and barrier effects, even without live microbes (10). In Caco-2 cell barrier models, the complete fermented cabbage matrix protected against cytokine-induced barrier damage better than single metabolites such as lactate, D-PLA and ILA, and these effects were not strictly dependent on microbial survival (47). Similarly, fermented vegetable brine and microbe-free supernatants showed comparable microbiota restructuring and barrier-tolerogenic activity (25). In patients with irritable bowel syndrome, unpasteurised and pasteurized sauerkraut led to similar symptom improvement (91). LFV consumption may also alter fecal metabolites more strongly than microbial composition, supporting functional gut microbiome modulation without durable colonization (92).

A common immune-mediated MGBA pathway is the modulation of mucosal barrier function and systemic inflammatory tone. Gut microbiota and microbial metabolites can influence mucosal immunity and systemic cytokine milieu, thereby affecting brain function and behavior (84). In humans, a high-fermented-food diet reduced multiple inflammatory markers while increasing microbiota diversity, although the intervention was not strictly LFV-specific (88). In preclinical work, fermented vegetable brine promoted tolerogenic barrier immunity, implying reduced pro-inflammatory signaling capacity from the gut (25).

Barrier modulation represents a closely related pathway (71). Strengthening epithelial tight junctions and the mucus layer can reduce the translocation of inflammatory luminal products, thereby altering systemic immune signaling (10). Fermented foods can strengthen intestinal barrier through metabolites, including SCFAs and organic acids (10, 15, 16, 93). LFVs provide lactate and fermentable plant substrates that resident microbes convert into SCFAs, including butyrate and propionate (10). SCFAs regulate barrier function and mucosal immune tone (94, 95). Propionate can increase satiety via gut hormones such as PYY and GLP-1, while butyrate supports colonocyte energy metabolism and epithelial integrity (94, 96).

LFVs are also discussed as food sources of neuroactive microbial metabolites (10, 12, 13). One example is gamma-aminobutyric acid (GABA), produced by LAB via glutamate decarboxylation (42). Orally ingested GABA has limited direct access to the brain (97). However, LFV-derived GABA can participate in peripheral enteric, immune and inflammatory signaling, including effects on gut motility and secretion (43). Thus, LFV-derived GABA should be interpreted as a peripheral neuroimmune and enteric signaling molecule, not as a direct CNS neurotransmitter (97, 98). Visceral signaling then propagates to the CNS indirectly via immune, endocrine and neural routes, including vagal afferents (10).

Changes in bowel habits and visceral sensitivity are MGBA-relevant because disorders of gut-brain interaction, such as irritable bowel syndrome (IBS), involve motility changes, visceral signaling, enteric nervous system activity, microbiota-immune interactions, and CNS processing (99). Sauerkraut consumption improved symptom severity in patients with IBS (91). While this was not a direct brain-outcome study, improvement in IBS-related symptoms supports the relevance to gut–brain sensory and immune signaling, including spinal and vagal afferent pathways (80, 99).

Vegetable fermentation also transforms phytochemicals and plant matrices, generating polyphenols and other small bioactive metabolites (13, 100). Some polyphenols and microbial metabolites can enter systemic circulation and may interact with the blood-brain barrier (101, 102). Evidence from broader polyphenol research suggests that selected polyphenols and their metabolites can cross the blood-brain barrier, modulate microglia and influence neurotrophic pathways, such as BDNF-related signaling involved in synaptic plasticity (11, 103–106). Experimental MGBA studies further show that individual microbial metabolites can have brain-region- and cell-type-specific effects in preclinical models (107). These data support metabolite-specific gut-brain mechanisms, although they are not yet LFV-specific.

Overall, direct evidence for LFV effects on the MGBA remains limited. While fermented foods, the gut microbiome, and psychobiological outcomes are increasingly investigated, LFV-specific mechanistic and clinical data remain scarce. Links between LFVs and MGBA-related outcomes should therefore be interpreted as mechanistically plausible routes deduced from broader fermented foods and MGBA research. Targeted mechanistic and human interventional studies are needed to clarify these associations.

## 4 The importance of the small intestine and enteroendocrine cells

Most human microbiome studies use fecal samples, which mainly reflect distal colonic conditions (108). However, food-derived microbes and metabolites travel through the mouth, stomach, duodenum, jejunum and ileum before reaching the colon. The small intestine is likely an important signaling site that differs markedly from the colon in nutrient availability, pH, oxygen exposure, transit time, immune architecture and enteroendocrine activity (109, 110). The ileum holds highly active immune monitoring structures such as Peyer's patches (111). It is likewise enriched in enteroendocrine signaling related to motility, appetite control and gut-brain communication (110, 111).

Enteroendocrine cells sense luminal nutrients, microbial metabolites and bile acids through G-protein-coupled receptors, including GPR41, GPR43, HCA-receptors and bile acid receptors. (112–114). They translate these signals into endocrine, paracrine, and neural pathways, releasing mediators such as GLP-1, PYY, cholecystokinin, serotonin, and ghrelin, thereby affecting appetite regulation, motility, secretion, glucose metabolism, autonomic activity, visceral signaling, and stress-related pathways (113–116). Specialized enteroendocrine subtypes, termed neuropod cells, form synapse-like connections with enteric neurons and vagal afferents, allowing fast transmission of luminal signals to neural pathways (112, 115–118).

These mechanisms may be relevant for LFVs because fermentation-derived metabolites may be sensed before extensive colonic transformation occurs (86). Spencer et al showed that sauerkraut brine altered fecal and caecal microbial composition, with the strongest changes observed in the ileum, where *Akkermansia muciniphila* and regulatory T cells were enriched (25). These data support the plausibility that LFV-derived compounds may influence enteroendocrine, neural, immune and barrier-related pathways before reaching the colon. However, direct human evidence for LFV-signaling in the small intestine is still limited.

## 5 Health outcomes of LFVs in humans

Existing reviews and intervention studies indicate possible benefits for gastrointestinal health, cardiometabolic risk factors and gut microbial function (10, 13). However, no human study has directly connected LFV-consumption to MGBA-measures. In patients with irritable bowel syndrome, daily sauerkraut consumption for six weeks improved symptom severity and altered gut microbial composition, with no significant difference between pasteurized and unpasteurised products (91). In healthy adults, LFV effects on gut microbial composition appear product- and person-dependent. A sauerkraut cross-over trial detected changes in individual bacterial species and heightened serum SCFA after pasteurized sauerkraut consumption (119).

Evidence from Kimchi studies is more extensive. A scoping review including 11 RCTs found ameliorations in serum lipid profiles and body-fat-related parameters (120). A systematic review and meta-analysis of intervention and prospective cohort studies found improvements in fasting glucose, triglycerides and blood pressure, with observational evidence linking higher kimchi intake to normal weight and lower risk of metabolic syndrome and cancer (121).

These preliminary findings support the health-improving effects of LFVs. Nevertheless, human studies linking LFVs to neurologic and psychological health are still scarce. Ongoing clinical research is beginning to address this gap by integrating microbiome, immune and patient-reported outcome measures (PROMs). A recently published RCT protocol investigates fermented vs. unfermented red cabbage effects on allergic rhinitis symptoms, immune markers, the gut microbiome, and PROMs for depression and anxiety in young adults (122).

On a final note, we would like to emphasize that LFVs can show considerable microbial and compositional variability, which may sometimes raise safety concerns for human consumption. Surveys of homemade and commercial LFVs generally found mature products free of major pathogens. However, considerable heterogeneity in acidity, LAB and yeast abundance, and salt content was observed, with occasional detection of potentially undesirable microorganisms (123, 124). In a challenge study in which pathogens where inoculated into sauerkraut, they remained viable during early fermentation, particularly when acidification was delayed (125). LFVs may also contain biogenic amines and high salt concentrations, which may pose further challenges for human consumption (126). Good hygiene and well-controlled fermentation conditions are therefore essential.

## 6 Future directions

Future human studies should connect LFV-mediated microbial and metabolic changes with immune, neuroendocrine and clinical outcomes. Relevant MGBA-endpoints may include intestinal barrier markers, salivary cortisol as HPA-axis marker, heart-rate variability as an autonomic proxy, neuroimmune cytokine panels, and neurocognitive and affective outcomes, including neuroimaging techniques. Additionally integrating food multi-omics could help identify LFV preparations with specific mechanistic properties. This direction is consistent with the concept of engineered LAB-fermentates as functional fermented foods with defined health-supporting properties (79, 81, 127).

Research on specific bacteria, bacteriocins, or bacteriophages in LFVs may yield important insights into pressing current problems such as antimicrobial resistance.

Another research avenue is to explore how agricultural methods influence LFV composition and possible health effects. Different cultivation systems shape plant-associated microbiomes. Raw plant material entering fermentation carries microbial signatures influenced by farming practices. This links soil, plants, plant fermentation, and the human gut microbiome (7).

LFVs may also serve as controllable complex model ecosystems for studying food-gut microbiome-host interactions. Fermented foods are dynamic ecological systems in which bacteria, fungi, bacteriophages, metabolites and environmental conditions interact, giving rise to emergent functions. Upcoming research could leverage this property to explore how external pressures, such as cultivation practices, fermentation conditions, or antibiotic exposure, influence ecosystem evolution, resilience, invasion resistance, recovery after perturbation, and transition toward dysfunctional states (17, 128).

Overall, LFVs remain an intriguing but underexplored area within fermented-food research. Future advances in our understanding of LFVs may help clarify their potential as targeted components of nutritional strategies for intestinal, immune and neuropsychological health.

## References

[1] Marco ML Sanders ME Gänzle M Arrieta MC Cotter PD De Vuyst L . The international scientific association for probiotics and prebiotics (ISAPP) consensus statement on fermented foods. Nat Rev Gastroenterol Hepatol. (2021) 18:196–208. doi: 10.1038/s41575-020-00390-5 33398112 PMC7925329

[2] Campbell-Platt G. Fermented foods — a world perspective. Food Res Int. (1994) 27:253–7. doi: 10.1016/0963-9969(94)90093-0

[3] Marco ML Heeney D Binda S Cifelli CJ Cotter PD Foligné B . Health benefits of fermented foods: microbiota and beyond. Curr Opin Biotechnol. (2017) 44:94–102. doi: 10.1016/j.copbio.2016.11.010 27998788

[4] Gänzle M. The periodic table of fermented foods: limitations and opportunities. Appl Microbiol Biotechnol. (2022) 106:2815–26. doi: 10.1007/s00253-022-11909-y 35412130

[5] Louw NL Lele K Ye R Edwards CB Wolfe BE. Microbiome assembly in fermented foods. Ann Rev Microbiol. (2023) 77:381–402. doi: 10.1146/annurev-micro-032521-041956 37713453

[6] Nemergut DR Schmidt SK Fukami T O'Neill SP Bilinski TM Stanish LF . Patterns and processes of microbial community assembly. Microbiol Mol Biol Rev. (2013) 77:342–56. doi: 10.1128/MMBR.00051-12 24006468 PMC3811611

[7] Wassermann B Müller H Berg G. An apple a day: which bacteria do we eat with organic and conventional apples? Front Microbiol. (2019) 10:1629. doi: 10.3389/fmicb.2019.01629 31396172 PMC6667679

[8] Lu Z Breidt F Plengvidhya V Fleming HP. Bacteriophage ecology in commercial sauerkraut fermentations. Appl Environ Microbiol. (2003) 69:3192–202. doi: 10.1128/AEM.69.6.3192-3202.2003 12788716 PMC161505

[9] Aslam H Green J Jacka FN Collier F Berk M Pasco J . Fermented foods, the gut and mental health: a mechanistic overview with implications for depression and anxiety. Nutr Neurosci. (2020) 23:659–71. doi: 10.1080/1028415X.2018.1544332 30415609

[10] Balasubramanian R Schneider E Gunnigle E Cotter PD Cryan JF. Fermented foods: harnessing their potential to modulate the microbiota-gut-brain axis for mental health. Neurosci Biobehav Rev. (2024) 158:105562. doi: 10.1016/j.neubiorev.2024.105562 38278378

[11] Bodur M Kocaadam-Bozkurt B Bozkurt O Aslan S Agagündüz D. Fermented foods and brain health: gut-brain axis mechanisms and clinical insights. J Nutr Biochem. (2026) 149:110195. doi: 10.1016/j.jnutbio.2025.110195 41297620

[12] Shawky E Surendran S El-Khair RMA. Fermented vegetables as a source of psychobiotics: a review of the evidence for mental health benefits. Probiotics Antimicrob Proteins. (2026) 18:1587–601. doi: 10.1007/s12602-025-10592-5 40402417 PMC12999709

[13] Wei L Van Beeck W Hanlon M DiCaprio E Marco ML. Lacto-fermented fruits and vegetables: bioactive components and effects on human health. Annu Rev Food Sci Technol. (2025) 16:289–314. doi: 10.1146/annurev-food-052924-070656 39805038

[14] Tyliszczak M Wiatrak B Danielewski M Szelag A Kucharska AZ Sozański T. Does a pickle a day keep Alzheimer's away? Fermented food in Alzheimer's disease: a review. Exp Gerontol. (2023) 184:112332. doi: 10.1016/j.exger.2023.112332 37967591

[15] Dimidi E Cox SR Rossi M Whelan K. Fermented foods: definitions and characteristics, impact on the gut microbiota and effects on gastrointestinal health and disease. Nutrients. (2019.) 11:1806. doi: 10.3390/nu11081806 31387262 PMC6723656

[16] Leeuwendaal NK Stanton C O'Toole PW Beresford TP. Fermented foods, health and the gut microbiome. Nutrients. (2022) 14:1527. doi: 10.3390/nu14071527 35406140 PMC9003261

[17] Park I Mannaa M. Fermented foods as functional systems: microbial communities and metabolites influencing gut health and systemic outcomes. Foods. (2025) 14:2292. doi: 10.3390/foods14132292 40647044 PMC12249102

[18] Yu AO Leveau JHJ Marco ML. Abundance, diversity and plant-specific adaptations of plant-associated lactic acid bacteria. Environ Microbiol Rep. (2020) 12:16–29. doi: 10.1111/1758-2229.12794 31573142

[19] Köberl M Erschen S Etemadi M White RA El-Arabi TF Berg G. Deciphering the microbiome shift during fermentation of medicinal plants. Sci Rep. (2019) 9:13461. doi: 10.1038/s41598-019-49799-2 31530872 PMC6748931

[20] Gaudioso G Weil T Marzorati G Solovyev P Bontempo L Franciosi E . Microbial and metabolic characterization of organic artisanal sauerkraut fermentation and study of gut health-promoting properties of sauerkraut brine. Front Microbiol. (2022) 13:929738. doi: 10.3389/fmicb.2022.929738 36312966 PMC9606823

[21] Pedersen MGB Søndergaard E Nielsen CB Johannsen M Gormsen LC Møller N . Oral lactate slows gastric emptying and suppresses appetite in young males. Clin Nutr. (2022) 41:517–25. doi: 10.1016/j.clnu.2021.12.032 35016146

[22] Anumudu CK Miri T Onyeaka H. Multifunctional applications of lactic acid bacteria: enhancing safety, quality, and nutritional value in foods and fermented beverages. Foods. (2024) 13:3714. doi: 10.3390/foods13233714 39682785 PMC11640447

[23] Zapaśnik A Sokołowska B Bryła M. Role of lactic acid bacteria in food preservation and safety. Foods. (2022) 11:1283. doi: 10.3390/foods11091283 35564005 PMC9099756

[24] Li X Yao Z Qian J Li H Li H. Lactate protects intestinal epithelial barrier function from dextran sulfate sodium-induced damage by GPR81 signaling. Nutrients. (2024) 16:582. doi: 10.3390/nu16050582 38474712 PMC10934655

[25] Spencer S Silva EGL Caffery EB Carter MM Culver RN Wang M . Fermented foods restructure gut microbiota and promote immune regulation via microbial metabolites. bioRxiv. (2022). doi: 10.1101/2022.05.11.490523

[26] Culp EJ Goodman AL. Cross-feeding in the gut microbiome: ecology and mechanisms. Cell Host Microbe. (2023) 31:485–99. doi: 10.1016/j.chom.2023.03.016 37054671 PMC10125260

[27] Wang Y Wu J Lv M Shao Z Hungwe M Wang J . Metabolism characteristics of lactic acid bacteria and the expanding applications in food industry. Front Bioeng Biotechnol. (2021) 9:612285. doi: 10.3389/fbioe.2021.612285 34055755 PMC8149962

[28] Gill PA Bogatyrev A van Zelm MC Gibson PR Muir JG. Delivery of acetate to the peripheral blood after consumption of foods high in short-chain fatty acids. Mol Nutr Food Res. (2021) 65:2000953. doi: 10.1002/mnfr.202000953 33377265

[29] Valdes DS So D Gill PA Kellow NJ. Effect of dietary acetic acid supplementation on plasma glucose, lipid profiles, and body mass index in human adults: a systematic review and meta-analysis. J Acad Nutr Dietet. (2021) 121:895–914. doi: 10.1016/j.jand.2020.12.002 33436350

[30] Mishra SP Karunakar P Taraphder S Yadav H. Free fatty acid receptors 2 and 3 as microbial metabolite sensors to shape host health: pharmacophysiological view. Biomedicines. (2020) 8:154. doi: 10.3390/biomedicines8060154 32521775 PMC7344995

[31] Annunziata G Arnone A Ciampaglia R Tenore GC Novellino E. Fermentation of foods and beverages as a tool for increasing availability of bioactive compounds. Focus on short-chain fatty acids. Foods. (2020) 9:999. doi: 10.3390/foods9080999 32722417 PMC7466228

[32] Hu R Zeng F Wu L Wan X Chen Y Zhang J . Fermented carrot juice attenuates type 2 diabetes by mediating gut microbiota in rats. Food Funct. (2019) 10:2935–46. doi: 10.1039/C9FO00475K 31070649

[33] Stiemsma LT Nakamura RE Nguyen JG Michels KB. Does consumption of fermented foods modify the human gut microbiota? J Nutr. (2020) 150:1680–92. doi: 10.1093/jn/nxaa077 32232406 PMC7330458

[34] Veiga P Pons N Agrawal A Oozeer R Guyonnet D Brazeilles R . Changes of the human gut microbiome induced by a fermented milk product. Sci Rep. (2014) 4:6328. doi: 10.1038/srep06328 25209713 PMC4160712

[35] Liu Y Hou Y Wang G Zheng X Hao H. Gut microbial metabolites of aromatic amino acids as signals in host–microbe interplay. Trends Endocrinol Metab. (2020) 31:818–34. doi: 10.1016/j.tem.2020.02.012 32284282

[36] Xu GC Zhang LL Ni Y. Enzymatic preparation of D-phenyllactic acid at high space-time yield with a novel phenylpyruvate reductase identified from Lactobacillus sp. CGMCC 9967. J Biotechnol. (2016) 222:29–37. doi: 10.1016/j.jbiotec.2015.12.011 26712480

[37] Yang X Li J Shi G Zeng M Liu Z. Improving 3-phenyllactic acid production of Lactobacillus plantarum AB-1 by enhancing its quorum-sensing capacity. J Food Sci Technol. (2019) 56:2605–10. doi: 10.1007/s13197-019-03746-1 31168142 PMC6525690

[38] Peters A Krumbholz P Jäger E Heintz-Buschart A Çakir MV Rothemund S . Metabolites of lactic acid bacteria present in fermented foods are highly potent agonists of human hydroxycarboxylic acid receptor 3. PLoS Genet. (2019). 15:e1008145. doi: 10.1371/journal.pgen.1008145 31120900 PMC6532841

[39] Ehrlich AM Pacheco AR Henrick BM Taft D Xu G Huda MN . Indole-3-lactic acid associated with Bifidobacterium-dominated microbiota significantly decreases inflammation in intestinal epithelial cells. BMC Microbiol. (2020) 20:357. doi: 10.1186/s12866-020-02023-y 33225894 PMC7681996

[40] Sakurai T Horigome A Odamaki T Shimizu T Xiao JZ. Production of Hydroxycarboxylic Acid Receptor 3 (HCA) Ligands by Bifidobacterium. Microorganisms. (2021) 9:2397. doi: 10.3390/microorganisms9112397 34835522 PMC8620054

[41] Wang A Guan C Wang T Mu G Tuo Y. Lactiplantibacillus plantarum-derived indole-3-lactic acid ameliorates intestinal barrier integrity through the AhR/Nrf2/NF-κB axis. J Agri Food Chem. (2024) 72:9236–46. doi: 10.1021/acs.jafc.4c01622 38597152

[42] Cui Y Miao K Niyaphorn S Qu X. Production of gamma-aminobutyric acid from lactic acid bacteria: a systematic review. Int J Mol Sci. (2020) 21:995. doi: 10.3390/ijms21030995 32028587 PMC7037312

[43] Auteri M Zizzo MG Serio R. GABA and GABA receptors in the gastrointestinal tract: from motility to inflammation. Pharmacol Res. 2015. 93:11–21. doi: 10.1016/j.phrs.2014.12.001 25526825

[44] Wawrzyniak M O'Mahony L Akdis M. Role of regulatory cells in oral tolerance. Allergy Asthma Immunol Res. (2017) 9:107–15. doi: 10.4168/aair.2017.9.2.107 28102055 PMC5266108

[45] Levan SR Stamnes KA Lin DL Panzer AR Fukui E McCauley K . Elevated faecal 12,13-diHOME concentration in neonates at high risk for asthma is produced by gut bacteria and impedes immune tolerance. Nat Microbiol. (2019) 4:1851–61. doi: 10.1038/s41564-019-0498-2 31332384 PMC6830510

[46] Park B Hwang H Chang JY Hong SW Lee SH Jung MY . Identification of 2-hydroxyisocaproic acid production in lactic acid bacteria and evaluation of microbial dynamics during kimchi ripening. Sci Rep. (2017) 7:10904. doi: 10.1038/s41598-017-10948-0 28883404 PMC5589888

[47] Wei L Marco ML. The fermented cabbage metabolome and its protection against cytokine-induced intestinal barrier disruption of Caco-2 monolayers. Appl Environ Microbiol. (2025) 91:e0223424. doi: 10.1128/aem.02234-24 40192297 PMC12093966

[48] Septembre-Malaterre A Remize F Poucheret P. Fruits and vegetables, as a source of nutritional compounds and phytochemicals: changes in bioactive compounds during lactic fermentation. Food Res Int. (2018) 104:86–99. doi: 10.1016/j.foodres.2017.09.031 29433787

[49] Langa S Peirotén Á Curiel JA de la Bastida AR Landete JM. Isoflavone metabolism by lactic acid bacteria and its application in the development of fermented soy food with beneficial effects on human health. Foods. (2023) 12:1293. doi: 10.3390/foods12061293 36981219 PMC10048179

[50] Michlmayr H Kneifel W. β-Glucosidase activities of lactic acid bacteria: mechanisms, impact on fermented food and human health. FEMS Microbiol Lett. (2014) 352:1–10. doi: 10.1111/1574-6968.12348 24330034

[51] Scheepens A Tan K Paxton JW. Improving the oral bioavailability of beneficial polyphenols through designed synergies. Genes Nutr. (2010) 5:75–87. doi: 10.1007/s12263-009-0148-z 19841960 PMC2820202

[52] Martinez-Villaluenga C Penas E Sidro B Ullate M Frias J Vidal-Valverde C. White cabbage fermentation improves ascorbigen content, antioxidant and nitric oxide production inhibitory activity in LPS-induced macrophages. LWT Food Sci Technol. (2012) 46:77–83. doi: 10.1016/j.lwt.2011.10.023

[53] Palani K Harbaum-Piayda B Meske D Keppler JK Bockelmann W Heller KJ . Influence of fermentation on glucosinolates and glucobrassicin degradation products in sauerkraut. Food Chem. (2016) 190:755–62. doi: 10.1016/j.foodchem.2015.06.012 26213035

[54] Adebo OA Gabriela Medina-Meza I. Impact of fermentation on the phenolic compounds and antioxidant activity of whole cereal grains: a mini review. Molecules. (2020) 25:927. doi: 10.3390/molecules25040927 32093014 PMC7070691

[55] Chen C Ai Q-d Wei Y-h. Potential role of hydroxytyrosol in neuroprotection. J Funct Foods. (2021) 82:104506. doi: 10.1016/j.jff.2021.104506

[56] D'Angelo C Franceschelli S Quiles JL Speranza L. Wide biological role of hydroxytyrosol: possible therapeutic and preventive properties in cardiovascular diseases. Cells. (2020) 9:1932. doi: 10.3390/cells9091932 32825589 PMC7565717

[57] D'Antuono I Bruno A Linsalata V Minervini F Garbetta A Tufariello M . Fermented apulian table olives: effect of selected microbial starters on polyphenols composition, antioxidant activities and bioaccessibility. Food Chem. (2018) 248:137–45. doi: 10.1016/j.foodchem.2017.12.032 29329836

[58] Pastor R Bouzas C Tur JA. Beneficial effects of dietary supplementation with olive oil, oleic acid, or hydroxytyrosol in metabolic syndrome: systematic review and meta-analysis. Free Radic Biol Med. (2021) 172:372–85. doi: 10.1016/j.freeradbiomed.2021.06.017 34153478

[59] Dogan S Koç TY Karadayi M. Effect of bacteriocins on the intestinal microbiota. Eurasian J Med. (2023) 55:165–9. doi: 10.5152/eurasianjmed.2023.23393 39128034 PMC11074934

[60] Alvarez-Sieiro P Montalbán-López M Mu D Kuipers OP. Bacteriocins of lactic acid bacteria: extending the family. Appl Microbiol Biotechnol. (2016) 100:2939–51. doi: 10.1007/s00253-016-7343-9 26860942 PMC4786598

[61] Zhang D Zhang J Kalimuthu S Liu J Song ZM He BB . A systematically biosynthetic investigation of lactic acid bacteria reveals diverse antagonistic bacteriocins that potentially shape the human microbiome. Microbiome. (2023) 11:91. doi: 10.1186/s40168-023-01540-y 37101246 PMC10134562

[62] Angelin J Kavitha M. Exopolysaccharides from probiotic bacteria and their health potential. Int J Biol Macromol. (2020) 162:853–65. doi: 10.1016/j.ijbiomac.2020.06.190 32585269 PMC7308007

[63] Laiño J Villena J Kanmani P Kitazawa H. Immunoregulatory effects triggered by lactic acid bacteria exopolysaccharides: new insights into molecular interactions with host cells. Microorganisms. (2016) 4:27. doi: 10.3390/microorganisms4030027 27681921 PMC5039587

[64] Salminen S Collado MC Endo A Hill C Lebeer S Quigley EMM . The international scientific association of probiotics and prebiotics (ISAPP) consensus statement on the definition and scope of postbiotics. Nat Rev Gastroenterol Hepatol. (2021) 18:649–67. doi: 10.1038/s41575-021-00440-6 33948025 PMC8387231

[65] Bersch KL DeMeester KE Zagani R Chen S Wodzanowski KA Liu S . Bacterial peptidoglycan fragments differentially regulate innate immune signaling. ACS Cent Sci. (2021) 7:688–96. doi: 10.1021/acscentsci.1c00200 34056099 PMC8155477

[66] Wells JM. Immunomodulatory mechanisms of lactobacilli. Microb Cell Fact. (2011) 10:S17. doi: 10.1186/1475-2859-10-S1-S17 21995674 PMC3231924

[67] Zheng D Liwinski T Elinav E. Interaction between microbiota and immunity in health and disease. Cell Res. (2020) 30:492–506. doi: 10.1038/s41422-020-0332-7 32433595 PMC7264227

[68] Knez E Kadac-Czapska K Grembecka M. Effect of fermentation on the nutritional quality of the selected vegetables and legumes and their health effects. Life. (2023) 13:655. doi: 10.3390/life13030655 36983811 PMC10051273

[69] Thompson HO Önning G Holmgren K Strandler HS Hultberg M. Fermentation of cauliflower and white beans with lactobacillus plantarum - impact on levels of riboflavin, folate, vitamin B, and amino acid composition. Plant Foods Hum Nutr. (2020) 75:236–42. doi: 10.1007/s11130-020-00806-2 32144644 PMC7266841

[70] Kiczorowski P Kiczorowska B Samolińska W Szmigielski M Winiarska-Mieczan A. Effect of fermentation of chosen vegetables on the nutrient, mineral, and biocomponent profile in human and animal nutrition. Sci Rep. (2022) 12:13422. doi: 10.1038/s41598-022-17782-z 35927577 PMC9352655

[71] Mukherjee A Breselge S Dimidi E Marco ML Cotter PD. Fermented foods and gastrointestinal health: underlying mechanisms. Nat Rev Gastroenterol Hepatol. (2024) 21:248–66. doi: 10.1038/s41575-023-00869-x 38081933

[72] Park WJ Kong SJ Park JH. Kimchi bacteriophages of lactic acid bacteria: population, characteristics, and their role in watery kimchi. Food Sci Biotechnol. (2021) 30:949–57. doi: 10.1007/s10068-021-00930-y 34395026 PMC8302715

[73] Yoon SS Barrangou-Poueys R Breidt F Klaenhammer TR Fleming HP. Isolation and characterization of bacteriophages from fermenting sauerkraut. Appl Environ Microbiol. (2002) 68:973–6. doi: 10.1128/AEM.68.2.973-976.2002 11823247 PMC126688

[74] White K Yu JH Eraclio G Dal Bello F Nauta A Mahony J . Bacteriophage-host interactions as a platform to establish the role of phages in modulating the microbial composition of fermented foods. Microbiome Res Rep. (2022) 1:3. doi: 10.20517/mrr.2021.04 38089066 PMC10714293

[75] Fazzino L Anisman J Chacón JM Heineman RH Harcombe WR. Lytic bacteriophage have diverse indirect effects in a synthetic cross-feeding community. ISME J. (2020) 14:123–34. doi: 10.1038/s41396-019-0511-z 31578469 PMC6908662

[76] Arroyo-López FN Romero-Gil V Bautista-Gallego J Rodríguez-Gómez F Jiménez-Díaz R García-García P . Potential benefits of the application of yeast starters in table olive processing. Front Microbiol. (2012). 3:161. doi: 10.3389/fmicb.2012.00161 22558000 PMC3338231

[77] Cheng Z Yang J Yan R Wang B Bai Y Miao Z . Interactive mechanism-guided microbial interaction dynamics in food fermentations: lactic acid bacteria and yeasts as a case example. Food Biosci. (2025) 68:106453. doi: 10.1016/j.fbio.2025.106453

[78] Holzer P. Gut signals and gut feelings: science at the interface of data and beliefs. Front Behav Neurosci. (2022) 16:929332. doi: 10.3389/fnbeh.2022.929332 35874652 PMC9296981

[79] Cryan JF O'Riordan KJ Cowan CSM Sandhu KV Bastiaanssen TFS Boehme M . The microbiota-gut-brain axis. Physiol Rev. (2019) 99:1877–2013. doi: 10.1152/physrev.00018.2018 31460832

[80] Bonaz B Bazin T Pellissier S. The vagus nerve at the interface of the microbiota-gut-brain axis. Front Neurosci. (2018) 12:49. doi: 10.3389/fnins.2018.00049 29467611 PMC5808284

[81] O'Riordan KJ Collins MK Moloney GM Knox EG Aburto MR Fülling C . Short chain fatty acids: microbial metabolites for gut-brain axis signalling. Mol Cell Endocrinol. (2022) 546:111572. doi: 10.1016/j.mce.2022.111572 35066114

[82] Erny D Hrabě de Angelis AL Jaitin D Wieghofer P Staszewski O David E . Host microbiota constantly control maturation and function of microglia in the CNS. Nat Neurosci. (2015) 18:965–77. doi: 10.1038/nn.4030 26030851 PMC5528863

[83] Braniste V Al-Asmakh M Kowal C Anuar F Abbaspour A Tóth M . The gut microbiota influences blood-brain barrier permeability in mice. Sci Transl Med. (2014) 6:263ra158. doi: 10.1126/scitranslmed.3009759 25411471 PMC4396848

[84] O'Riordan KJ Moloney GM Keane L Clarke G Cryan JF. The gut microbiota-immune-brain axis: therapeutic implications. Cell Rep Med. (2025) 6:101982. doi: 10.1016/j.xcrm.2025.101982 40054458 PMC11970326

[85] Roselli M Natella F Zinno P Guantario B Canali R Schifano E . Colonization ability and impact on human gut microbiota of foodborne microbes from traditional or probiotic-added fermented foods: a systematic review. Front Nutr. (2021) 8:689084. doi: 10.3389/fnut.2021.689084 34395494 PMC8360115

[86] Caffrey EB Sonnenburg JL Devkota S. Our extended microbiome: the human-relevant metabolites and biology of fermented foods. Cell Metab. (2024) 36:684–701. doi: 10.1016/j.cmet.2024.03.007 38569469 PMC12220828

[87] Noh H Jang HH Kim G Zouiouich S Cho SY Kim HJ . Taxonomic composition and diversity of the gut microbiota in relation to habitual dietary intake in korean adults. Nutrients. (2021) 13:366. doi: 10.3390/nu13020366 33530330 PMC7912254

[88] Wastyk HC Fragiadakis GK Perelman D Dahan D Merrill BD Yu FB . Gut-microbiota-targeted diets modulate human immune status. Cell. (2021) 184:4137–53.e14. doi: 10.1016/j.cell.2021.06.019 34256014 PMC9020749

[89] Berding K Bastiaanssen TFS Moloney GM Boscaini S Strain CR Anesi A . Feed your microbes to deal with stress: a psychobiotic diet impacts microbial stability and perceived stress in a healthy adult population. Mol Psychiatry. (2023) 28:601–10. doi: 10.1038/s41380-022-01817-y 36289300 PMC9908549

[90] Chu H Mazmanian SK. Innate immune recognition of the microbiota promotes host-microbial symbiosis. Nat Immunol. (2013) 14:668–75. doi: 10.1038/ni.2635 23778794 PMC4109969

[91] Nielsen ES Garnas E Jensen KJ Hansen LH Olsen PS Ritz C . Lacto-fermented sauerkraut improves symptoms in IBS patients independent of product pasteurisation–a pilot study. Food Funct. (2018) 9:5323–35. doi: 10.1039/C8FO00968F 30256365

[92] Guse K Sharma A Weyenberg E Davison S Ma Y Choi Y . Regular consumption of lacto-fermented vegetables has greater effects on the gut metabolome compared with the microbiome. Gut Microbiome. (2023) 4:e11. doi: 10.1017/gmb.2023.9 39295909 PMC11406409

[93] Terpou A Dahiya D Nigam PS. Evolving dynamics of fermented food microbiota and the gut microenvironment: strategic pathways to enhance human health. Foods. (2025) 14:2361. doi: 10.3390/foods14132361 40647113 PMC12249264

[94] Ney LM Wipplinger M Grossmann M Engert N Wegner VD Mosig AS. Short chain fatty acids: key regulators of the local and systemic immune response in inflammatory diseases and infections. Open Biol. (2023) 13:230014. doi: 10.1098/rsob.230014 36977462 PMC10049789

[95] Peng L Li ZR Green RS Holzman IR Lin J. Butyrate enhances the intestinal barrier by facilitating tight junction assembly via activation of AMP-activated protein kinase in Caco-2 cell monolayers. J Nutr. (2009) 139:1619–25. doi: 10.3945/jn.109.104638 19625695 PMC2728689

[96] Jyoti Dey P. Mechanisms and implications of the gut microbial modulation of intestinal metabolic processes. NPJ Metab Health Dis. (2025) 3:24. doi: 10.1038/s44324-025-00066-1 40604123 PMC12441142

[97] Hepsomali P Groeger JA Nishihira J Scholey A. Effects of oral gamma-aminobutyric acid (GABA) administration on stress and sleep in humans: a systematic review. Front Neurosci. (2020) 14:923. doi: 10.3389/fnins.2020.00923 33041752 PMC7527439

[98] Boonstra E de Kleijn R Colzato LS Alkemade A Forstmann BU Nieuwenhuis S. Neurotransmitters as food supplements: the effects of GABA on brain and behavior. Front Psycho. (2015) 6:1520. doi: 10.3389/fpsyg.2015.01520 26500584 PMC4594160

[99] Mayer EA Ryu HJ Bhatt RR. The neurobiology of irritable bowel syndrome. Mol Psychiatry. (2023) 28:1451–65. doi: 10.1038/s41380-023-01972-w 36732586 PMC10208985

[100] Yang F Chen C Ni D Yang Y Tian J Li Y . Effects of fermentation on bioactivity and the composition of polyphenols contained in polyphenol-rich foods: a review. Foods. (2023) 12:3315. doi: 10.3390/foods12173315 37685247 PMC10486714

[101] Figueira I Garcia G Pimpão RC Terrasso AP Costa I Almeida AF . Polyphenols journey through blood-brain barrier towards neuronal protection. Sci Rep. (2017) 7:11456. doi: 10.1038/s41598-017-11512-6 28904352 PMC5597593

[102] Filosa S Di Meo F Crispi S. Polyphenols-gut microbiota interplay and brain neuromodulation. Neural Regen Res. (2018) 13:2055–9. doi: 10.4103/1673-5374.241429 30323120 PMC6199944

[103] Johnson SL Kirk RD DaSilva NA Ma H Seeram NP Bertin MJ. Polyphenol microbial metabolites exhibit gut and blood–brain barrier permeability and protect murine microglia against LPS-induced inflammation. Metabolites. (2019) 9:78. doi: 10.3390/metabo9040078 31010159 PMC6523162

[104] Li R Zhou Y Zhang S Li J Zheng Y Fan X. The natural (poly)phenols as modulators of microglia polarization via TLR4/NF-κB pathway exert anti-inflammatory activity in ischemic stroke. Eur J Pharmacol. (2022) 914:174660. doi: 10.1016/j.ejphar.2021.174660 34863710

[105] Hunt T Pontifex MG Vauzour D. (Poly)phenols and brain health – beyond their antioxidant capacity. FEBS Lett. (2024) 598:2949–62. doi: 10.1002/1873-3468.14988 39043619 PMC11665953

[106] Lu B Nagappan G Lu Y. BDNF and synaptic plasticity, cognitive function, and dysfunction. Handbook Experiment Pharmacol. (2014) 220:223–50. doi: 10.1007/978-3-642-45106-5_9 24668475

[107] Needham BD Funabashi M Adame MD Wang Z Boktor JC Haney J . A gut-derived metabolite alters brain activity and anxiety behaviour in mice. Nature. (2022) 602:647–53. doi: 10.1038/s41586-022-04396-8 35165440 PMC9170029

[108] Shalon D Culver RN Grembi JA Folz J Treit PV Shi H . Profiling the human intestinal environment under physiological conditions. Nature. (2023) 617:581–91. doi: 10.1038/s41586-023-05989-7 37165188 PMC10191855

[109] Jensen BAH Heyndrickx M Jonkers D Mackie A Millet S Naghibi M . Small intestine vs. colon ecology and physiology: why it matters in probiotic administration. Cell Rep Med. (2023) 4:101190. doi: 10.1016/j.xcrm.2023.101190 37683651 PMC10518632

[110] Yilmaz B Macpherson AJ. Delving the depths of ‘terra incognita' in the human intestine — the small intestinal microbiota. Nat Rev Gastroenterol Hepatol. (2025) 22:71–81. doi: 10.1038/s41575-024-01000-4 39443711

[111] Park JI Cho SW Kang JH Park TE. Intestinal peyer's patches: structure, function, and in vitro modeling. Tissue Eng Regen Med. (2023) 20:341–53. doi: 10.1007/s13770-023-00543-y 37079198 PMC10117255

[112] Barton JR Londregan AK Alexander TD Entezari AA Covarrubias M Waldman SA. Enteroendocrine cell regulation of the gut-brain axis. Front Neurosci. (2023) 17:1272955. doi: 10.3389/fnins.2023.1272955 38027512 PMC10662325

[113] Latorre R Sternini C De Giorgio R Greenwood-Van Meerveld B. Enteroendocrine cells: a review of their role in brain-gut communication. Neurogastroenterol Motil. (2016) 28:620–30. doi: 10.1111/nmo.12754 26691223 PMC4842178

[114] Worthington JJ Reimann F GribbleFM. Enteroendocrine cells-sensory sentinels of the intestinal environment and orchestrators of mucosal immunity. Mucosal Immunol. (2018) 11:3–20. doi: 10.1038/mi.2017.73 28853441

[115] Kaelberer MM Buchanan KL Klein ME Barth BB Montoya MM Shen X . A gut-brain neural circuit for nutrient sensory transduction. Science. (2018) 361:eaat5236. doi: 10.1126/science.aat5236 30237325 PMC6417812

[116] Ye L Bae M Cassilly CD Jabba SV Thorpe DW Martin AM . Enteroendocrine cells sense bacterial tryptophan catabolites to activate enteric and vagal neuronal pathways. Cell Host Microbe. (2021) 29:179–196.e9. doi: 10.1016/j.chom.2020.11.011 33352109 PMC7997396

[117] Bohórquez DV Shahid RA Erdmann A Kreger AM Wang Y Calakos N . Neuroepithelial circuit formed by innervation of sensory enteroendocrine cells. J Clin Investig. (2015) 125:782–6. doi: 10.1172/JCI78361 25555217 PMC4319442

[118] Kaelberer MM Rupprecht LE Liu WW Weng P Bohórquez DV. Neuropod cells: the emerging biology of gut-brain sensory transduction. Annu Rev Neurosci. (2020) 43:337–53. doi: 10.1146/annurev-neuro-091619-022657 32101483 PMC7573801

[119] Schropp N Bauer A Stanislas V Huang KD Lesker TR Bielecka AA . The impact of regular sauerkraut consumption on the human gut microbiota: a crossover intervention trial. Microbiome. (2025) 13:52. doi: 10.1186/s40168-024-02016-3 39940045 PMC11817299

[120] Song E Ang L Lee HW Kim M-S Kim YJ Jang D . Effects of kimchi on human health: a scoping review of randomized controlled trials. J Ethnic Foods. (2023) 10:7. doi: 10.1186/s42779-023-00173-8

[121] Ahn S Darooghegi Mofrad M Nosal BM Chun OK Joung H. Effects of fermented kimchi consumption on anthropometric and blood cardiometabolic indicators: a systematic review and meta-analysis of intervention studies and prospective cohort studies. Nutr Rev. (2025) 83:e1441–57. doi: 10.1093/nutrit/nuae167 39545368

[122] Ngoumou GB Ngandeu Schepanski S Blakeslee SB Diedering A Twal E Raue SL . Effects of fermented versus unfermented red cabbage on symptoms, immune response, inflammatory markers and the gut microbiome in young adults with allergic rhinoconjunctivitis: a randomised controlled trial protocol. BMJ Open. (2026) 16:e115290. doi: 10.1136/bmjopen-2025-115290 41857857 PMC13007187

[123] Thierry A Madec MN Chuat V Bage AS Picard O Grondin C . Microbial communities of a variety of 75 homemade fermented vegetables. Front Microbiol. (2023) 14:1323424. doi: 10.3389/fmicb.2023.1323424 38163080 PMC10757351

[124] Vermeersch M Mayr C Maes A Wieme AD De Meulenaer B Vandamme P . Microbiological survey of spontaneous vegetable fermentations: a food safety perspective. Int J Food Microbiol. (2026) 446:111541. doi: 10.1016/j.ijfoodmicro.2025.111541 41330088

[125] Niksic M Niebuhr SE Dickson JS Mendonca AF Koziczkowski JJ Ellingson JL. Survival of Listeria monocytogenes and Escherichia coli O157:H7 during sauerkraut fermentation. J Food Protect. (2005) 68:1367–74. doi: 10.4315/0362-028X-68.7.1367 16013372

[126] Swider O Roszko MŁ Wójcicki M Szymczyk K. Biogenic amines and free amino acids in traditional fermented vegetables—dietary risk evaluation. J Agri Food Chem. (2020) 68:856–68. doi: 10.1021/acs.jafc.9b05625 31891502

[127] Mathur H Beresford TP Cotter PD. Health benefits of lactic acid bacteria (LAB) fermentates. Nutrients. (2020) 12:1679. doi: 10.3390/nu12061679 32512787 PMC7352953

[128] Zhou H Tang L Fenton KA Song X. Exploring and evaluating microbiome resilience in the gut. FEMS Microbiol Ecol. (2025) 101:fiaf046. doi: 10.1093/femsec/fiaf046 40302016 PMC12065411

